# A Dual sgRNA Approach for Functional Genomics in *Arabidopsis thaliana*

**DOI:** 10.1534/g3.118.200046

**Published:** 2018-06-08

**Authors:** Laurens Pauwels, Rebecca De Clercq, Jonas Goossens, Sabrina Iñigo, Clara Williams, Mily Ron, Anne Britt, Alain Goossens

**Affiliations:** * Ghent University, Department of Plant Biotechnology and Bioinformatics, 9052 Ghent, Belgium; † VIB Center for Plant Systems Biology, 9052 Ghent, Belgium; ‡ UC Davis, Department of Plant Biology, Davis, CA 95616, US

**Keywords:** *Arabidopsis thaliana*, genome engineering, genome editing, RNA-guided nuclease, alt-EJ, polymerase theta, CRISPR/Cas9

## Abstract

Reverse genetics uses loss-of-function alleles to interrogate gene function. The advent of CRISPR/Cas9-based gene editing now allows the generation of knock-out alleles for any gene and entire gene families. Even in the model plant *Arabidopsis thaliana*, gene editing is welcomed as T-DNA insertion lines do not always generate null alleles. Here, we show efficient generation of heritable mutations in Arabidopsis using CRISPR/Cas9 with a workload similar to generating overexpression lines. We obtain for several different genes Cas9 null-segregants with bi-allelic mutations in the T2 generation. While somatic mutations were predominantly generated by the canonical non-homologous end joining (cNHEJ) pathway, we observed inherited mutations that were the result of synthesis-dependent microhomology-mediated end joining (SD-MMEJ), a repair pathway linked to polymerase θ (PolQ). We also demonstrate that our workflow is compatible with a dual sgRNA approach in which a gene is targeted by two sgRNAs simultaneously. This paired nuclease method results in more reliable loss-of-function alleles that lack a large essential part of the gene. The ease of the CRISPR/Cas9 workflow should help in the eventual generation of true null alleles of every gene in the Arabidopsis genome, which will advance both basic and applied plant research.

The precise introduction of a DNA double-strand break (DSB) in a plant genome can now be accomplished through a variety of techniques (Baltes and Voytas 2015). However, the advent of Clustered Regularly Interspaced Short Palindromic Repeats (CRISPR)/CRISPR associated protein 9 (CRISPR/Cas9)-based technology has brought reliable gene editing (GE) within the reach of non-specialized molecular biology labs. The power of CRISPR/Cas9 compared to predecessor techniques lies in both a consistent high efficiency and a simple two-component design. A generic nuclease, Cas9, is guided to a target DNA sequence (protospacer) by associating with an artificial single guide RNA (sgRNA) (Jinek *et al.* 2012). Changing the typically 20 nucleotide long target-specific spacer sequence in the sgRNA is sufficient for redirecting the RNA-guided engineered nuclease to another genomic locus. In addition, several sgRNAs with different targets can be co-expressed allowing for multiplexing as exemplified in *Arabidopsis thaliana* by targeting of the PYRABACTIN RESISTANCE1-LIKE (PYL) family of abscisic acid receptor genes (Zhang *et al.* 2016) or the GOLVEN family (Peterson *et al.* 2016).

DSBs are readily recognized by the plant cell and repaired. The non-homologous end-joining (NHEJ) pathway results in imprecise repair, producing small insertions and/or deletions (indels) at the cut site (Knoll *et al.* 2014). In Arabidopsis, one base pair (bp) insertions (+1) are usually observed in somatic cells (Fauser *et al.* 2014, Feng *et al.* 2014). Alternative EJ (alt-EJ) uses a molecularly distinct mechanism and microhomologies flanking the cut site to guide repair. Also known as microhomology-mediated end joining (MMEJ), alt-EJ often detected by relatively larger deletions that are generated (Knoll *et al.* 2014). A model of synthesis-dependent MMEJ (SD-MMEJ) was proposed that can explain different MMEJ repair outcomes, including the presence of templated insertions at the junction (Yu and McVey, 2010, Khodaverdian *et al.* 2017). NHEJ-mediated indel-formation is used to generate loss-of-function mutants. If the indel causes a frame-shift, a non-functional truncated protein can be translated, and/or a premature stop codon will trigger nonsense-mediated decay (NMD) causing organized mRNA degradation by the cell (Popp and Maquat 2016).

CRISPR/Cas9 technology has been established for Arabidopsis and is continuously being developed further (Feng *et al.* 2013, Mao *et al.* 2013, Fauser *et al.* 2014, Feng *et al.* 2014, Ma *et al.* 2015, Wang *et al.* 2015, Osakabe *et al.* 2016, Tsutsui and Higashiyama 2017, Zhang *et al.*, 2016, Denbow *et al.* 2017, Peterson *et al.* 2016). Reports using CRISPR/Cas9 in Arabidopsis are emerging that are not technology-focused, but rather limited in number taking into account the widespread use of this model organism, the short generation time and its ease of transformation (Gao *et al.* 2015, Ning *et al.* 2015, Xin *et al.* 2016, Zhang *et al.* 2016, Guseman *et al.* 2017, Li *et al.* 2017, Lu *et al.* 2018, Ritter *et al.* 2017, Durr *et al.* 2018). The difficulties of using CRISPR/Cas9 to generate mutants in Arabidopsis have been attributed to the unique floral dip system of transformation in which inflorescences of T0 plants are infected with *Agrobacterium tumefaciens*. Primary transformants (T1) are derived via this process from a transformed egg cell (Bechtold *et al.* 2000). Chimerism, *i.e.*, the presence of at least 3 different alleles, points to Cas9 activity at later stages during somatic growth. This indicates that the mutation did not occur within the egg cell or zygote, but rather after the first cell division. Furthermore, even when mutations are detected in T1 somatic cells, often WT alleles are retrieved once the CRISPR/Cas9 T-DNA has been segregated away (Wang *et al.* 2015, Durr *et al.* 2018). This can be attributed to gene editing efficiency, *i.e.*, the percentage of cells not WT, as the limited number of cells that make up the germ line have to be mutated for heritability. Recently, low activity of SpCas9 at 21° has been suggested to be causal as there was an increase in CRISPR/Cas9 mutagenesis in both somatic and germline mutations at 37° (LeBlanc *et al.* 2018).

Here, we report and quantify high editing efficiencies in T1 somatic cells and inheritance of NHEJ-repaired alleles in Arabidopsis. In T1 somatic cells we observe mostly single base pair insertions, which are likely the result of cNHEJ. In contrast, inherited mutations for three different sgRNAs showed alt-EJ repair outcomes of which two consistent with criteria for SD-MMEJ. Our workflow allows us to obtain Cas9 null-segregants with bi-allelic mutations in the T2 generation. Moreover, it is compatible with a dual sgRNA approach, leading to deletion of gene fragments and greater confidence in producing loss-of-function alleles.

## Materials and Methods

### Design of sgRNAs

In general, sgRNAs were selected for specificity using CRISPR-P (http://crispr.hzau.edu.cn/CRISPR/, Lei *et al.* 2014), taking into account predicted on-target efficiencies using sgRNAscorer (https://crispr.med.harvard.edu/sgRNAScorer/, Chari *et al.* 2015). An updated overview of estimated sgRNA parameters by CRISP-OR (http://crispor.tefor.net/, Haeussler *et al.* 2016) can be found in Table 1.

**Table 1:** sgRNA parameters used in this study

Name	Type^1^		Protospacer + PAM	Specificity^2^	Chari^3^	Doench^4^	Mor.-Mateos^5^	Observed efficiency^6^	Efficiency median^7^	Chimerism^8^
VQ33-42	trugRNA	G-N18	GATGAGGAGATATTATCTG AGG	95	79	72	57	75,0	92,2	5,4
VQ33-38	starts with G	G-N19	GCCTTAACGTATTGATCATT AGG	96	2	36	28	84,4	94,6	5,9
VQ33-1	starts with G	G-N19	GGGTCATCGTTGCTTCTCAG TGG	100	58	66	56	75,4	94,2	4,5
VQ19-6	starts with G	G-N19	GGGACTGTTAAGTGCAAGCT TGG	99	28	48	45	34,4	19,6	3,5
VQ19-34	starts with G	G-N19	GCGGAGAGTCTGGAGATCTT GGG	99	60	44	50	66,1	82,3	7,6
GRXS17-4	starts with G	G-N19	GACCTTCGAGCCGAGCTCGG AGG	100	99	64	58	67,3	83,2	4,2
GLB3-3	starts with G	G-N19	GATAAGGCATCGGTGTTAAG CGG	100	88	62	56	77,7	96,1	6,6
JAM2-109	starts with G	G-N19	GGAGATTTGGTTCTCTGTTG GGG	97	31	48	53	88,6	97,7	3,4
JAM2-140	extra G	G-N20	TATTGCAGAGAGCCTAAAGA AGG	96	80	56	36	26,4	4,5	2,5
GRXS17-133	extra G	G-N20	CTTGATAACTTGCGCCAGAG CGG	84	86	62	57	NA	NA	NA
GRXS17-67	extra G	G-N20	ATTATGGAGCTAAGTGAGAG TGG	98	87	63	28	NA	NA	NA
WRKY20-201	extra G	G-N20	ACTTCCCAAAATGACTCCAG AGG	100	97	69	64	NA	NA	NA
WRKY20-39	starts with G	G-N19	GTATGGCTGCACAAGAAGAA AGG	96	90	54	42	NA	NA	NA

^1^ , type of sgRNA depending on the position of the starting guanine nucleotide. ^2^, CRISPOR specificity score (0-100). ^3^, predicted efficiency score (0-100) by Chari *et al.* (2015). ^4^, predicted efficiency score (0-100) by Doench *et al.* (2014). ^5^, predicted efficiency score (0-100) by Moreno-Mateos *et al.* (2015). ^6^, observed efficiency (percentage of cells not WT) as the average efficiency indicated by TIDE T1 seedlings. ^7^, median efficiency indicated by TIDE T1 seedlings. ^8^, chimerism indicated as the average number of alleles present ≥ 1% in a T1 plant. NA, not applicable.

### Cloning of CRISPR/Cas9 constructs

CRISPR/Cas9 constructs were cloned as previously described (Figure S1, Fauser *et al.* 2014, Ritter *et al.* 2017). Briefly, for each guide sequence, two complementary oligos with 4bp overhangs (Supplementary Table S1) were annealed and inserted via a cut-ligation reaction with BbsI (Thermo) and T4 DNA ligase (Thermo) in a Gateway ENTRY sgRNA shuttle vector. This is either pEN-C1.1 (Fauser *et al.* 2014) for single sgRNA constructs, or pMR217 (L1-R5) and pMR218 (L5-L2) (Ritter *et al.* 2017) for the dual sgRNA approach. The 5′ overhang already contains the G initiation nucleotide of the AtU6-26 polIII promoter. Next, using a Gateway LR reaction (ThermoFisher), one or two sgRNA modules were then combined with pDE-Cas9 (Basta, Fauser *et* al 2014) or pDE-Cas9Km (pMR169, Ritter *et al.* 2017) to yield the final expression clone.

### Plant transformation

Expression clones were introduced in the Agrobacterium strain C58C1 (pMP90) using electroporation, which was used to transform Arabidopsis using the floral dip method (Clough and Bent 1998).

### Plant Material and Growth Conditions

*Arabidopsis thaliana* Col-0 were grown at 21° under long day (16-h light/8-h dark) conditions. Rapid selection of seeds with kanamycin and phosphinothricin (BASTA) selection was performed as described (Harrison *et al.* 2006).

### Selection of CRISPR/Cas9 mutants

Typically, 16 kanamycin- or BASTA-resistant T1 plants are selected *in vitro* and transferred to a growth room. After 14 days, a single leaf is harvested, and genomic DNA prepared using Edwards buffer (Edwards *et al.* 1991). Next, 5 µl template gDNA was used as a template in a standard 20 µl volume PCR reaction using GoTaq (Promega) with the supplied Green GoTaq Reaction Buffer. For single sgRNA constructs, the amplicon was treated with ExoSAP-IT (Thermo) and sequenced by standard capillary sequencing at the VIB Genomics Core Facility (https://corefacilities.vib.be/gsf). Quantitative sequence trace data were decomposed using TIDE (https://tide.nki.nl/) using standard settings, except for the indel size range, which was set on the maximum (50). Primers for TIDE were designed using Primer3 (http://bioinfo.ut.ee/primer3-0.4.0/) using standard parameters. Approximately 700 bp asymmetrically surrounding the Cas9 cut site was amplified. The amplification primer at 200 bp from the site was used for sequencing.

For each independent T1 line, approximately 64 T2 seeds were selected on either BASTA or kanamycin. Resistant *vs.* sensitive seedlings were analyzed using a chi-squared test and lines presumably having a single T-DNA locus continued. Typically, 15 seedlings of the most promising line (highest T1 efficiency, expected segregation) were grown on non-selective media and genotyped for the presence of the T-DNA locus using Cas9-specific primers (Table S1). Cas9 null-segregants are then analyzed for modifications at the locus of interest. The most promising plants are then propagated to T3, in which absence of Cas9 and presence of the mutation/deletion is confirmed by PCR and sequencing.

### Amplicon subcloning

For confirmation of TIDE spectra, the PCR amplicon was cut from gel, purified using GeneJET PCR purification kit (Thermo Scientific) and cloned into pJET1.2 using the CloneJET PCR cloning kit (Thermo Scientific). Individual clones were sequenced using capillary electrophoresis.

### RT-qPCR

Seedlings were grown in the same conditions as in Iñigo *et al.* (2016). Seedlings were frozen in liquid nitrogen and total RNA was extracted using RNeasy plant mini kit (Qiagen) and DNAse I (Promega) treatment. Next, 1 µg of RNA was used for cDNA synthesis using iScript kit (Bio-Rad). qRT-PCR was performed on a LightCycler 480 system (Roche) using the Fast Start SYBR Green I PCR mix (Roche) with three biological repeats and three technical repeats. Data were analyzed using the second derivative maximum method and relative expression levels were determined using the comparative cycle threshold method.

### Data and reagent availability

Vectors and plant lines are available upon request. Primer sequences are provided in Supplemental Table S1. Accession numbers of the genes used in this study: GRXS17, AT4G04950; VQ19/MVQ4, AT3G15300; VQ33/MVQ3, AT5G53830; WRKY20, AT4G26640; WRKY2, AT5G56270; JAM2/bHLH13, AT1G01260; GLB3, AT4G32690. T-DNA lines used: grxs17-1, SALK_021301; wrky2-1, SALK_020399. All supplemental material available at Figshare: https://doi.org/10.25387/g3.6455840.

## Results

### High gene editing efficiency in T1 somatic tissue

**Figure 1:**
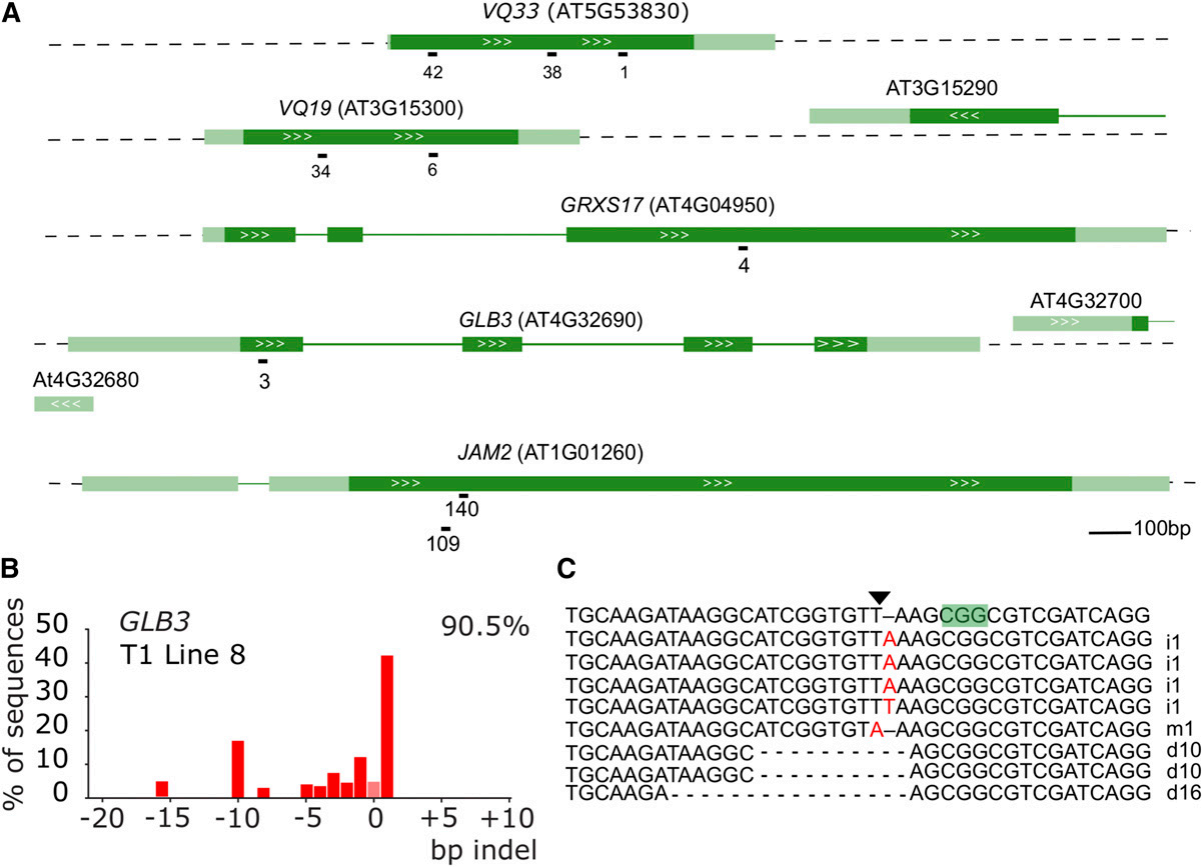
CRISPR/Cas9-induced somatic mutations in T1 Arabidopsis plants. A, genomic structure of the targeted genes and location of the sgRNAs. Dark green boxes designate exons; light green boxes, UTRs; solid lines, introns; white arrows gene orientation. sgRNA numbers are arbitrary identifiers. B, example result of a TIDE analysis. A leaf of a T1 plant expressing a CRISPR/Cas9 construct targeting *GLB3* was used to prepare genomic DNA. The targeted region was amplified by PCR and sequenced using standard Sanger sequencing. TIDE software was used to visualize the indel spectrum and estimate overall editing efficiency (top right corner). Bars indicate the number of sequences with a given indel size. Pink bar (indel size of zero) represents WT or base substitution alleles. C, Verification of TIDE using sequencing of individual amplicon subclones. The PAM is highlighted in green, the triangle points to the Cas9 cut site. i, insertion, d, deletion, m, mutation are followed with the number of bases involved.

The vector pDE-Cas9 has successfully been used for gene editing (GE) in Arabidopsis (Fauser *et al.* 2014). It contains an Arabidopsis codon-optimized SpCas9 sequence, driven by the *Petroselinum crispum* Ubiquitin4-2 promoter (pPcUBI). As kanamycin resistance is used more often in our lab, both in Arabidopsis and in tomato, we used pDE-Cas9Km (Ritter *et al.* 2017) in which the basta resistance cassette in pDE-Cas9 is replaced with *nptII* (Figure S1). In order to evaluate these vectors, we initially designed nine sgRNAs targeting five genes of interest: *JASMONATE ASSOCIATED MYC2 LIKE 2* (*JAM2*, Sasaki-Sekimoto *et al.* 2013), *VQ19* and *VQ33* (Jing and Lin 2015), *HEMOGLOBIN 3* (*GLB3*) and *GLUTAREDOXIN S17* (*GRXS17*) (Nagels Durand *et al.* 2016). sgRNAs were designed to minimize possible off-target activity (Lei *et al.* 2014), and when possible predicted sgRNA efficiencies were taken into account (Chari *et al.* 2015). An updated overview of estimated sgRNA parameters by CRISP-OR (http://crispor.tefor.net/, Haeussler *et al.* 2016) can be found in Table 1. Although it is currently unknown if the models for sgRNA efficiency, based on empirical data from metazoan cells holds true in plants, we anticipate that at least some sgRNA sequence parameters will be similar as CRISPR/Cas9 is a fully heterologous system. To better ensure the generation of loss-of-function alleles, sgRNAs were preferably chosen in the 5′ end of the first exon (Figure 1). In the case of *JAM2*, we specifically designed two sgRNAs that targeted the sequence encoding the JAZ interaction domain (JID) (Fernández-Calvo *et al.* 2011).

The sgRNA cloning procedure (Figure S1A) uses the type II restriction enzyme BbsI and utilizes a 5′ ATTG overhang of which the G serves as the first nucleotide of the sgRNA when transcribed by the polymerase III promoter AtU6-26. Most sgRNAs were of the GN19-type with the 5′ G being the first transcribed base of a 20-bp long guide sequence. One sgRNA, JAM2-140, was of the GN20-type. An extra 5′ G or GG attached to the sgRNA should not hinder efficiency (Cho *et al.* 2014). Another sgRNA VQ33-42, was a GN18-type. Truncated sgRNAs (tru-gRNAs) down to a 17bp guide sequence have been shown to be as efficient as 20bp guides in human cells (Fu *et al.* 2014).

For each single sgRNA construct, approximately 15 T1 Arabidopsis plants were selected on basta or kanamycin respectively. One of the first true leaves was harvested for genomic DNA extraction. A region spanning the predicted cut site was amplified by PCR and the amplicon sequenced by traditional Sanger sequencing. Arabidopsis CRISPR/Cas9 T1 plants are typically chimeric, defined as having at least three different alleles for a locus (Feng *et al.* 2014). Different cell files showed different indels in both alleles after NHEJ-mediated repair, leading to a range of detectable indels in a single leaf and a complex chromatogram. The quantitative sequence trace data were therefore decomposed using the Tracking of Indels by DEcomposition (TIDE) software (https://tide.nki.nl/) (Brinkman *et al.* 2014). This results in an estimation of overall editing efficiency (percentage of cells not WT) and the spectrum and frequency of the dominant indel types (See Figure 1B for an example for *GLB3*). Subcloning of amplicons followed by sequencing yielded similar profiles (Figure 1C). Furthermore, examination of genomic DNA of different leaves yielded comparable but not identical patterns (Figure S2).

**Figure 2:**
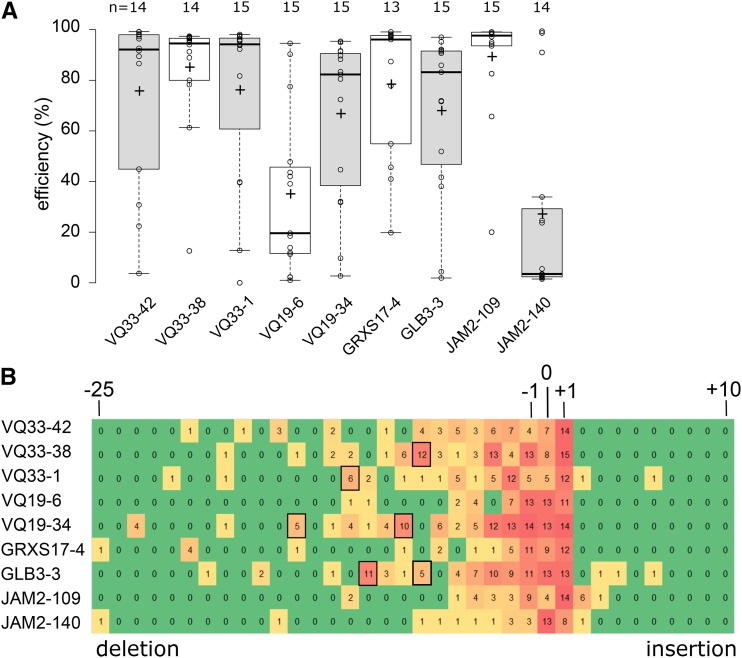
High gene editing efficiency in Arabidopsis T1 generation. A, boxplots showing TIDE estimated editing efficiencies for up to 15 T1 plants for nine different sgRNAs. +, mean; horizontal line, median; open circles, individual data points. B, heat map showing the number of T1 plants with at least 1% estimated frequency of an indel of a given size. Boxed are larger deletions (> 6 bp) observed in 5 or more T1 plants.

All but one sgRNA had high editing efficiencies with the median efficiency being higher than 80% (Figure 2A). Notably, VQ33-38, the sgRNA predicted by all three algorithms to have the worst efficiency (Table S2) had one of the highest efficiencies *in planta*. Next, we used the data generated, to investigate chimerism in the T1 plants. The most frequently observed mutation is a 1 bp insertion, followed by deletions of increasing size (Figure 2B). Large insertions were very uncommon. However, depending on the sgRNA larger deletions of a particular size were often observed. Potentially this is related to MMEJ, whereby regions of microhomology help initiate polymerase Q repair by annealing of single-stranded DNA overhangs (Black *et al.* 2016, Shen *et al.* 2017). In summary, we show high rates of CRISPR/Cas9 mutagenesis in Arabidopsis T1 somatic tissue for most tested sgRNAs and that TIDE is a robust method to evaluate sgRNA efficiency.

### Inheritance of mutations

**Figure 3:**
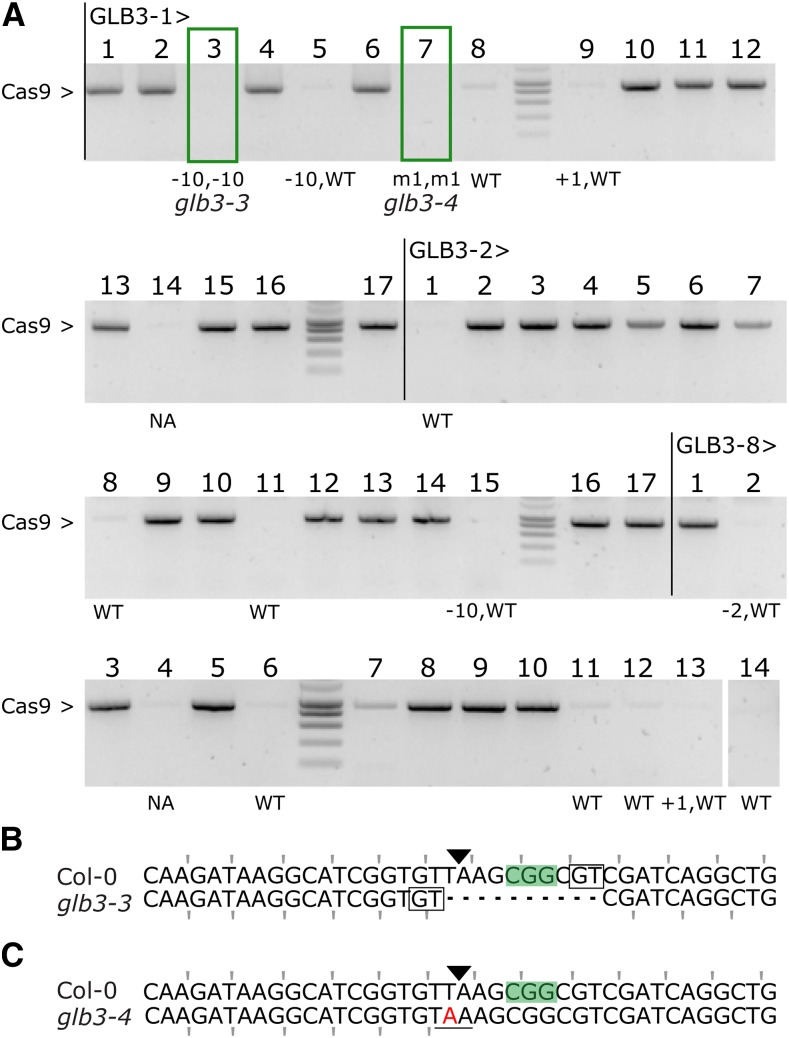
Inheritance of CRISPR/Cas9 mutations. A, PCR amplification of the Cas9 transgene in T2 seedlings from 3 independent GLB3 lines: -1, -2 and -8. Genotypes for all Cas9 null-segregants were estimated using TIDE. NA, not assayed; WT, wild-type; m1, 1bp substitution. Boxed plants were continued. B-C, Sequence alignment of the targeted locus for Col-0 and glb3-3 (B, Line 1, plant 3) or glb3-4 (C, Line 1, plant 7). PAM is highlighted, the Cas9 cut site indicated with a triangle, and microhomology boxed. Mutated bases are in red, deleted bases replaced by a dash. The reading frame is marked. The stop codon generated by the mutation is underlined.

Focusing on *GLB3*, we investigated the heritability of mutations after selfing and selected for T2 progeny that had lost the T-DNA (Cas9 null-segregants). First, we identified three T1 lines with a single T-DNA locus by segregation analysis of the kanamycin resistance marker in T2 seedlings. Of these three lines we germinated 14 to 17 seedlings on soil, prepared genomic DNA and genotyped using Cas9 specific primers to identify null-segregants (Figure 3A). The genomic DNA of these plants was re-used to amplify the target site and sequencing data were analyzed using TIDE to identify genotypes at the target locus. All 15 tested null-segregants were found to be non-chimeric: 8 were WT, 5 heterozygous and 2 were homozygous. Hence, inherited mutations were present in the T2 progeny of all three independent T1 lines. Although we only detected the desired homo-allelic Cas9 null-segregants in the progeny of one T1 line, heterozygous alleles will lead to the desired genotypes in the next generation. An outcome also overrepresented in T1 somatic mutations for *GLB3*, and frequently observed in the inherited mutations from independent events was a 10 bp deletion, indicative of alt-EJ (Figure 3B). Lastly, we identified a heritable T to A substitution which led to a single nucleotide variation (SNV) and here results in a premature stop codon (Figure 3B and 3C). This occurs when a single bp deletion is followed by a single bp insertion, an event very rarely observed for CRISPR/Cas9 (Kim *et al.* 2017). In conclusion, the pDE-Cas9 vectors allow for efficient and inheritable genome editing in Arabidopsis with the possibility of producing transgene free homo-allelic mutants in the T2 generation.

### Isolation of a new grxs17 CRISPR allele

**Figure 4:**
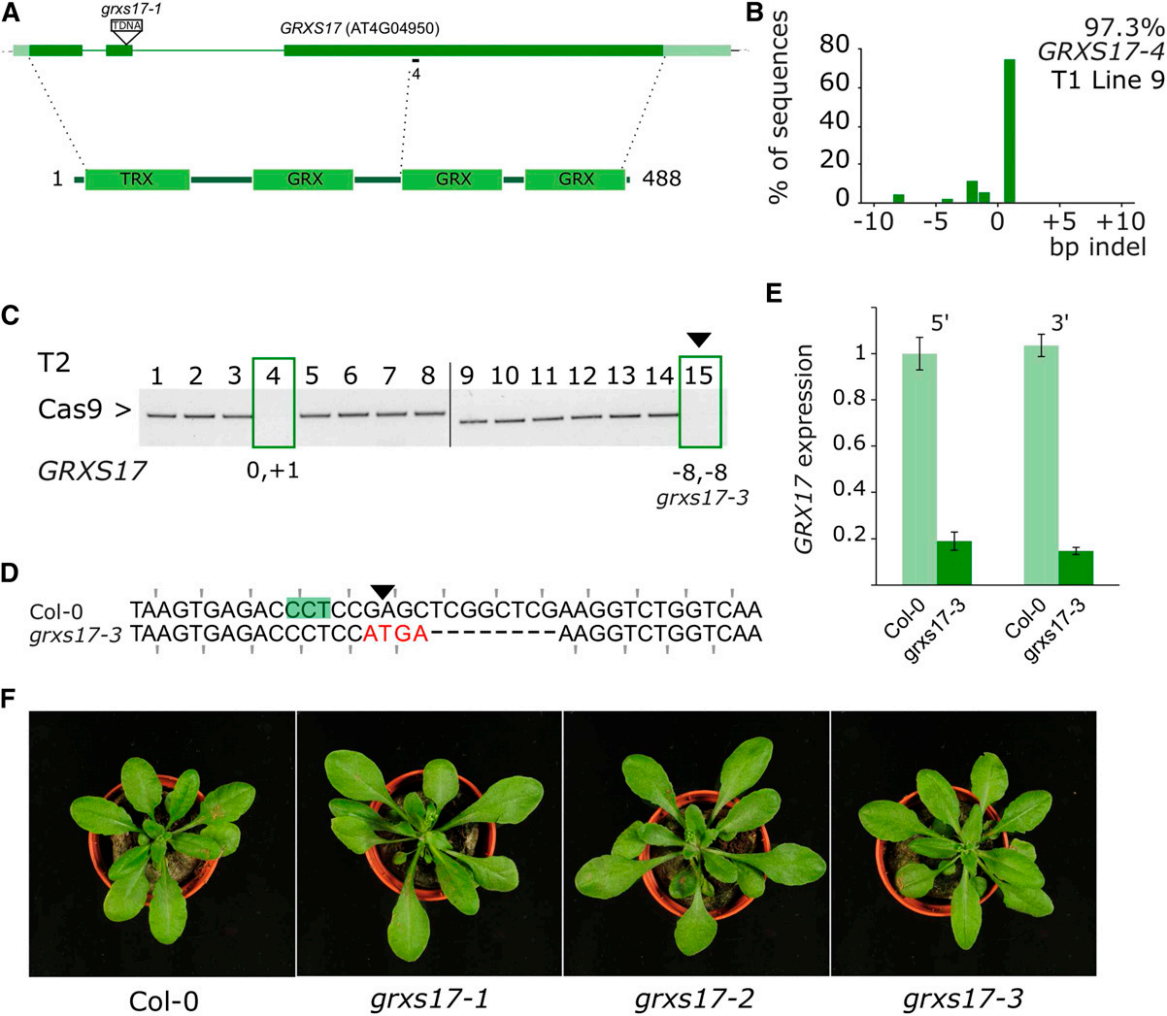
Generation and analysis of the *grxs17-3* allele. A, Gene and protein structure of GRXS17. The location of the *grxs17-1* T-DNA and the sgRNA used in this study are indicated. Dark green boxes designate exons; light green boxes, UTRs; solid lines, introns. TRX, thioredoxin domain; GRX, glutaredoxin domain. B, TIDE analysis of T1 line 9. Genomic DNA was PCR amplified and sequenced. The indel spectrum is visualized with an estimated overall efficiency and the frequency of each indel. C, PCR amplification of the Cas9 transgene. Null-segregants are boxed and the continued plant marked with a triangle. TIDE estimated genotypes for *GRXS17* are given for the null segregants. D, Sequence alignment of the targeted locus for Col-0 and grxs17-3 (Line 9, plant 15). PAM is highlighted, the Cas9 cut site indicated with a triangle. Mutated bases are in red, deleted bases replaced by an en dash. The reading frame is marked. E, *GRXS17* gene expression analyzed by RT-qPCR. Expression relative to Col-0 is plotted using primers annealing both at the 5′ and the 3′ of the transcript and the mutation. F, rosette phenotypes of Col-0, the T-DNA insertion line *grxs17-1*, the antisense line *grxs17-2* and the *grxs17-3* CRISPR allele.

Previously we characterized in detail two independent knock-out alleles of *GRXS17*, a gene encoding a component of the FeS cluster assembly pathway (Iñigo *et al.* 2016). The allele *grxs17-1* (SALK_021301) contains a T-DNA in the second exon (Figure 4A), whereas the *grxs17-2* allele expresses an antisense construct (Cheng *et al.* 2011).

A T1 parental line described above that showed high editing efficiency (97.3%) in somatic tissue and had a single T-DNA locus was identified (Figure 4B). Two Cas9 null-segregants of the T2 progeny were genotyped using TIDE (Figure 4C). This yielded the *grxs17-3* allele that was predicted to have a (-8,-8) genotype. Inspection of the sequence in T3 plants revealed an additional 4 base pair insertion, nevertheless leading to loss of the reading frame (Figure 4D). Using RT-qPCR, we could observe strong downregulation (∼80%) of the entire *GRXS17* transcript (Figure 4E). This is probably the result of nonsense-mediated decay (NMD), a process triggering mRNA degradation in case a premature stop codon is present (Popp and Maquat 2016). However, as there is no exon-exon boundary 3′ of the premature stop codon, this can be a case of exon-junction complex (EJC)-independent NMD, wherein NMD is triggered by a long 3′ UTR (Fatscher *et al.* 2014). Remarkably, the elongated leaf developmental phenotype present in both *grxs17-1* and *grxs17-2* was not visible in *grxs17-3* (Figure 4F). GRXS7 is a multidomain protein with an N-terminal thioredoxin (TRX) domain followed by three glutaredoxin (GRX) domains (Figure 4A). The human GRX3 ortholog has only 2 GRX domains, whereas the yeast Grx3/Grx4 orthologs have only one GRX domain (Couturier *et al.* 2015). We hypothesize that the *grxs17-3* allele is not a null allele and possibly expresses a C-terminally truncated GRXS17 protein with a functional TRX and GRX domain.

### A dual sgRNA approach for gene deletions

Choice of the sgRNA target site is pivotal in generating a reliable knock-out. Genes can contain alternative start codons, have alternative first exon usage, exon skipping and/or C-terminally truncated proteins and therefore might still be partially functional as exemplified above. In-depth knowledge on the gene structure, transcript and protein is therefore advisable. However, in many cases this information is not complete. Therefore, we examined in Arabidopsis a dual sgRNA approach in which two sgRNAs target the same gene to remove a large segment (Chen *et al.* 2014, Zhang *et al.* 2016, Ordon *et al.* 2017, Durr *et al.* 2018).

**Figure 5:**
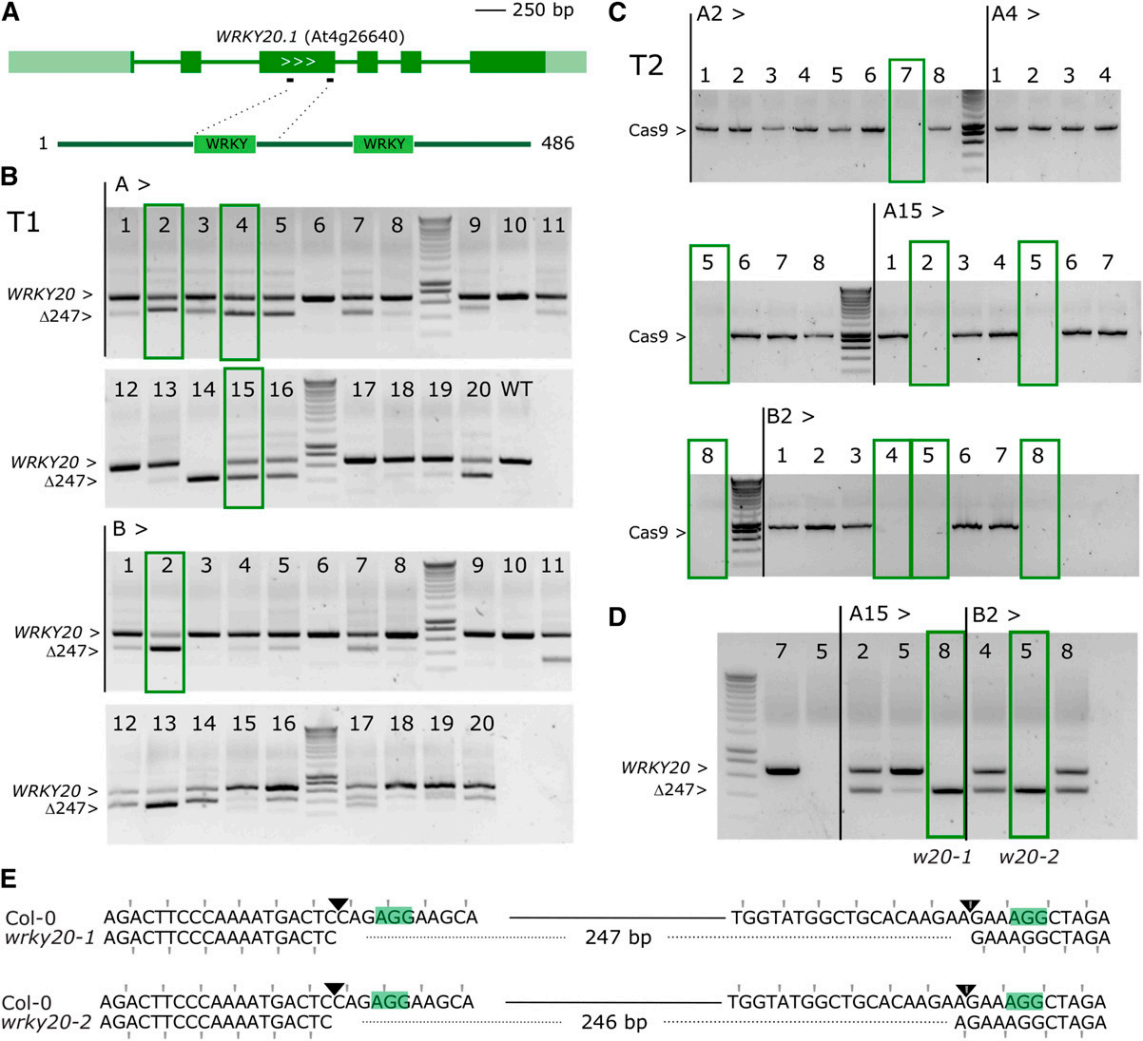
*WRKY20* dual sgRNA approach. A, genomic structure of *WRKY20* and location of the sgRNAs. Dark green boxes designate exons; light green boxes, UTRs; solid lines, introns. B, PCR analysis of T1 lines. Leaf genomic DNA of 2 batches (A and B) of 20 chimeric T1 plants was PCR amplified. The expected size of the WT *WRKY20* amplicon is indicated as well as the expected size of the deletion of 247 bp between Cas9 cut sites. Four continued T1 lines having one T-DNA locus are highlighted with green boxes. C, Cas9 PCR for the four continued lines in T2 generation. Putative Cas9 null-segregants are indicated with green boxes. D, Cas9 null-segregants were genotypes for WRKY20. The selected lines A15-8 (*wrky2-1 wrky20-1*) and B2-5 (*wrky2-1 wrky20-2*) are boxed. E, Sequence alignment of the simultaneously targeted loci for Col-0 and alleles *wrky20-1* and *wrky20-2*. PAMs are highlighted, the Cas9 cut sites indicated with triangles. Deleted bases are indicated with dashed lines. The reading frame is marked.

Using a MultiSite Gateway based sgRNA multiplexing approach we previously described (Ritter *et al.* 2017) we co-expressed two sgRNAs in pDE-Cas9Km. We used this method to target the gene encoding the transcription factor WRKY20, which is closely related to WRKY2, with two sgRNAs. For the latter, a characterized T-DNA insertion mutant *wrky2-1* is available representing a strong loss-of-function or null allele (Ueda *et al.* 2011). We transformed the *wrky2-1* background with a dual sgRNA construct for *WRKY20*, predicted to remove a 247 bp fragment encoding the first WRKY protein domain in addition to putting the remainder of the sequence out of frame (Figure 5A). Without any phenotypic selection, we applied the same workflow as before. We selected four independent T1 lines showing high levels of the expected deletion and containing a single T-DNA locus (Figure 5B). For each line, one or more null-segregants were identified in T2 (Figure 5C) and genotyped for the *WRKY20* locus. Of seven Cas9 null-segregants successfully genotyped, two plants were homozygous for the expected deletion, three heterozygous and two wild-type (Figure 5D). Sequence analysis of two homozygous deletion mutants showed that *wrky2-1 wrky20-1* (plant A15-8) had the predicted 247 bp deletion, whereas the other allele *wrky2-1 wrky20-2* (plant B2-5) only had a 246 bp segment deleted, possibly restoring the reading frame (Figure 5E). This shows that a dual sgRNA approach for deleting gene fragments is feasible with relatively few numbers of genotyped plants.

Next, we combined two sgRNAs targeting *VQ33* (VQ33-42 and VQ33-1) that displayed high efficiency when tested individually (Figure 2). Working together, they are predicted to remove a fragment of 459 bp, virtually removing the *VQ33* coding sequence (Figure S3A). We proceeded with the same workflow as for *WRKY20* (Figure S3B-D). Out of four Cas9 null-segregants, two were homozygous for the expected gene fragment deletion, one heterozyogous and one WT. The allele *vq33-1* (plant 11-8), albeit it had an extra 1 bp insertion, still led to a 458 bp out-of-frame deletion (Figure S3E).

In summary, we established a straightforward dual sgRNA approach to obtain plants homozygous for relatively large deletions of gene fragments in the T2 generation in *Arabidopsis thaliana*.

### grxs17-4 confirms the grxs17-1 developmental phenotype

**Figure 6:**
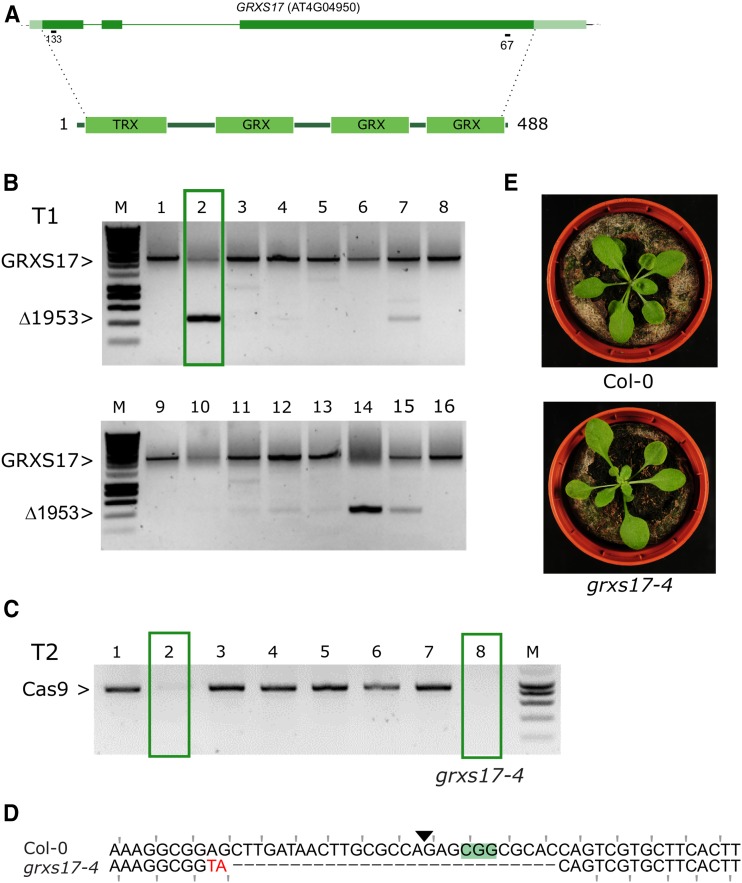
A dual sgRNA approach for *GRXS17*. A, genomic structure of *GRXS17* and location of the sgRNAs. Dark green boxes designate exons; light green boxes, UTRs; solid lines, introns. B, PCR analysis of T1 lines. Leaf genomic DNA of 16 chimeric T1 plants was PCR amplified. The expected size of the WT *GRXS17* amplicon is indicated as well as the expected size of the deletion of 1953 bp between Cas9 cut sites. One T1 line having one T-DNA locus that was continued is highlighted with a green box. C, Cas9 PCR for 8 T2 CRISPR plants. Putative Cas9 null-segregants are indicated with green boxes. D, Sequence alignment of the sequence surrounding the 5′ sgRNA site for Col-0 and *grxs17-4* (Line 2, plant 8). PAM is highlighted, the Cas9 cut site indicated with a triangle. Mutated bases are in red, deleted bases replaced by an en dash. The reading frame is marked. E, representative rosette phenotypes of WT Col-0 (top) and *grxs17-4* (bottom).

Next, we tried the dual sgRNA approach for *GRXS17*. We targeted the first sgRNA (GRXS17-133) at the 5′ end and the second sgRNA (GRXS17-67) at the 3′ end of the gene to remove 1953 bp and *GRXS17* almost entirely (Figure 6A). The *GRXS17* locus was amplified for sixteen independent T1 plants using primers spanning the expected deletion. In comparison with *VQ33* and *WRKY20*, only two plants clearly showed bands of the expected size for the predicted deletion (Figure 6B). Two identified Cas9-null segregants (Figure 6C) did not show the expected large deletion, but instead an indel was found at the first sgRNA site in the first exon leading to a frameshift (Figure 6D). The indel remarkably was a 30bp deletion combined with a 2bp insertion. We named this allele *grxs17-4*. Confirming our hypothesis that *grxs17-3* is indeed not a null allele, *grxs17-4* showed the leaf phenotype of *grxs17-1* and *grxs17-2* (Iñigo *et al.* 2016, Figure 6E). In conclusion, in the event the dual sgRNA approach does not yield the designed gene fragment deletion, each individual sgRNA may lead to useful alleles.

### Inherited mutations generated by synthesis-dependent MMEJ

**Figure 7:**
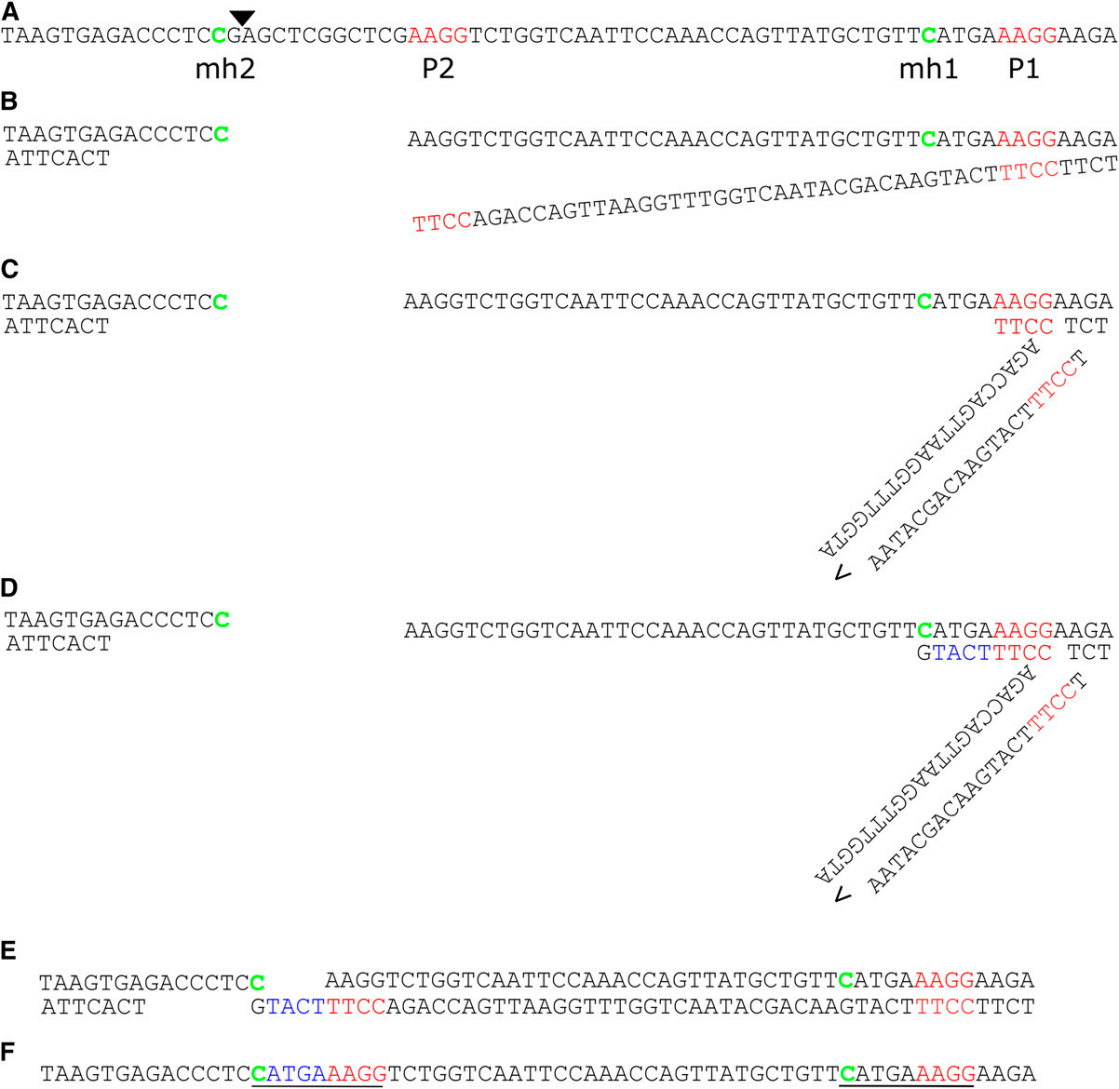
g*rxS17-3* is explained by loop out SD-MMEJ. A, *GRXS17* wild-type sequence surrounding the Cas9 cut site, which is indicated with a triangle. Primer repeat (P) regions are indicated in red with P2 break-proximal. Microhomology repeats (MH) are indicated in green. B, End resection by 5′-3′ nuclease activity and unwinding by helicase activity. C, loop formation by P1-P2 basepairing. D, templated elongation by polymerase activity. E, unwinding. F, annealing of mh2 with mh1 templated complimentary overhang. G, observed repair product in *grxs17-3*. Inserted nucleotides in blue, resulting direct repeat underlined.

It struck us that three of the final mutant alleles (*glb3-3*, *grxs17-3*, *grxs17-4*) we generated with a single sgRNA showed hallmarks of repair by alt-EJ. However, atypical for MMEJ, additional insertions were observed for *grxs17-3* and *grxs17-4*. Therefore, we analyzed the repair outcomes with criteria for SD-MMEJ, a model proposed for polymerase theta(PolQ)-mediated repair which also explains templated insertions (Khodaverdian *et al.* 2017). In this model, ends are resected at the DSB, after which microhomology regions called “primer repeat” (P1+P2) anneal via loops or hairpins to a region within 30 bp of the break. PolQ presumably then elongates the strand until another microhomology (MH1) is synthesized that has a counterpart (MH2) at the other side of the DSB, which then anneal to repair the break (Khodaverdian *et al.* 2017). In case for *grxs17-3* and *grxs17-4* we discovered that insertions could have been templated by neighboring sequences (Figure 7 and Figure S4). In both cases, a primer repeat was present upstream of the cut site, which after ‘loop out’ formation was elongated with 5 and 3 bp respectively. In both cases the microhomology repeat only consisted out of a single base, which nevertheless fits criteria for SD-MMEJ (Khodaverdian *et al.* 2017). A direct repeat can be observed (mh+insert+p) of 9 and 7 bp respectively. In summary, the model for SD-MMEJ is capable of explaining the observed inherited alt-EJ-associated mutations. This suggests a role for PolQ in DSB-repair in the Arabidopsis germline.

## Discussion

### Efficient CRISPR/Cas9 gene editing in Arabidopsis

The CRISPR/Cas9 technology shows promise to speed up reverse genetics experiments in Arabidopsis. Here we demonstrate efficient recovery of Cas9-free Arabidopsis mutants using single and double sgRNA constructs in the T2 generation without phenotypic selection. Previous negative experiences with CRISPR/Cas9 have been attributed to the weak activity of the 35S promoter in germ-line cells (Wang *et al.* 2015) or low activity of SpCas9 at 21° (LeBlanc *et al.* 2018). The promoter used here, PcUBI, is expressed widely, but detailed expression in germ-line cells has not yet been studied (Kawalleck *et al.* 1993). Other vector elements have been reported to play a role such as the vector backbone (Mao *et al.* 2016), Cas9 coding sequence (Johnson *et al.* 2015) and the terminator sequence (Wang *et al.* 2015). We did not observe any obvious differences using either *nptII* or *bar* as selection markers. Systematic analysis of all vector parameters is now achievable using modular cloning systems, which might allow identification of the best combinations (Vazquez-Vilar *et al.* 2016).

We consider the workflow presented here as already an acceptable workload comparable to the routine generation of overexpression lines (Figure S5). Nonetheless, several improvements have recently been developed. For example, a fluorescent marker for identification of transgenic T1 seeds has been reported (Tsutsui and Higashiyama 2017) and also cloned into pDE-Cas9 for CRISPR/Cas9 in *Camelina sativa* (Morineau *et al.* 2017, Durr *et al.* 2018). When Cas9 is driven with a promoter active in the egg cell, non-chimeric homozygous or bi-allelic mutants can already be retrieved in the T1 generation, although Cas9 null-segregants also only appear in T2 (Wang *et al.* 2015, Yan *et al.* 2015, Mao *et al.* 2016, Eid *et al.* 2016). Finally, applying 37° heat treatments in the vegetative phase may improve SpCas9 activity and would be compatible with our workflow (LeBlanc *et al.* 2018).

### TIDE as a useful tool to study mutations

Efficiency of CRISPR/Cas9 also clearly depends on the choice of sgRNA, although all sgRNAs tested in this study were active to some degree. Several models have been constructed to predict on-target editing efficiency based on the sgRNA primary sequence and on-target efficiency data from metazoans (Doench *et al.* 2014, Moreno-Mateos *et al.* 2015). Due to the lack of sufficient data, no plant-specific design models are currently available. As previously reported (Ordon *et al.* 2017), we did not observe any obvious correlation between these predictions and our observed efficiencies in Arabidopsis. It is unclear why this is the case for a heterologous system such as CRISPR/Cas9. Therefore - for the time being - we continue to take into account metazoan models when designing plant sgRNAs. It has been suggested to pre-screen sgRNAs in protoplasts (Li *et al.* 2014). Given the ease of Arabidopsis transformation via floral dip, we conclude from this study that designing several sgRNAs for the same target and testing somatic mutations in T1 might be an equally rapid method to identify efficient sgRNAs, while simultaneously obtaining the desired mutants.

Several methods have been used to study CRISPR/Cas9-induced mutations, most importantly cleaved amplified polymorphic sequence (CAPS), T7 endonuclease, next-generation sequencing and high-resolution melting curve analysis (Denbow *et al.* 2017). The method used here, TIDE (Brinkman *et al.* 2014), has several advantages. First, it does not require a restriction enzyme site overlapping the Cas9 cut site as in CAPS. Second, it allows the starting genomic DNA to be relatively impure allowing for more economic DNA extraction methods compared to T7-based assays. Third, it uses standard capillary Sanger sequencing that can be readily performed for even a single sample. Fourthly, it can provide an insight in the indel spectrum of mosaics similar to next-generation sequencing as well as providing an idea of overall efficiencies. These TIDE efficiencies are likely an underestimation. For example, TIDE is unable to detect rare SNVs as observed for *glb3-4*. The *grxs17-3* allele also revealed that mutations can be more complex than predicted by TIDE: a predicted 8 bp deletion was actually a 12 bp deletion combined with a 4 bp insertion.

### Know your target gene

The absence of the typical *grxs17* phenotype in the CRISPR allele *grxs17-3* is an example of how it is important to study independent alleles made with either different sgRNAs or with other methods when interpreting phenotypes of CRISPR/Cas9-generated alleles as knock-out effects. A seemingly widespread, but only recently discovered phenomenon is conditional alternative promoter selection, resulting in alternative N-termini (Ushijima *et al.* 2017). This makes it difficult to predict if indels at the beginning of the first annotated exon will affect all protein isoforms in all conditions. When sufficient information is available, especially on alternative transcripts and protein domain structures, sgRNA target sites can be chosen to maximize the chance of a complete knock-out as a result of an indel mutation at that site. Additionally, one may disrupt the gene more dramatically by removing a larger gene fragment using a dual sgRNA approach. The use of CRISPR/Cas9 for gene deletion has been pioneered in mammalian systems (Chen *et al.* 2014, Zhou *et al.* 2014, Ran *et al.* 2013, Canver *et al.* 2014). In Arabidopsis, a dual sgRNA approach for gene deletion was reported by Zhao *et al.* (2016) and Ordon *et al.* (2017). In Zhao *et al.* homozygous deletion mutants were obtained for the *AtMIR827a* and *AtMIR169a* loci in the T2 or T3 generation, respectively. The size of the deletion and efficiency seem to correlate inversely in mammalian cells (Canver *et al.* 2014) and plants (Ordon *et al.* 2017). Similarly, when attempting to cut out a 1953 kb fragment in *GRXS17*, it failed to be inherited, while clearly being present in T1 somatic cells. In contrast, 247 bp and 459 bp fragment deletions were easily obtainable for *WRKY20* and *VQ33* respectively. Therefore, while deleting whole genes might be tempting, it is more practical targeting genes with two sgRNAs in the 5′ coding sequence. This has the additional advantage, that when one sgRNA has a low efficiency, the construct will still yield potential knock-out mutations at the other sgRNA site. It has been proposed from work in tomato protoplasts that in most cases when a single sgRNA is used, NHEJ results in perfect repair and therefore using two sgRNAs could be more efficient to obtain mutants (Čermák *et al.* 2017). Finally, the double-sgRNA approach has an advantage of easy visual genotyping of mutants based on amplicon lengths.

### New alleles for GRXS17

*GRXS17* encodes the Arabidopsis ortholog of human *GRX3/PICOT* and yeast *Grx3/Grx4*. Although a role for GRXS17 in iron-sulfur cluster assembly is conserved in all of these organisms, plant-specific functions for GRXS17 are apparent (Iñigo *et al.* 2016, Knuesting *et al.* 2017). Interestingly, AtGRXS17, HsGRX3 and ScGrx3/4 differ in the number of GRX domains that are C-terminal of the TRX domain with three, two and one domain present, respectively. The new *grxs17-3* allele presented here might have residual expression of a truncated GRXS17 with only one GRX domain—similar to ScGrx3/4—and could therefore be helpful in studying plant-specific GRXS17 roles. More detailed molecular and phenotypical analysis of this allele and the other alleles generated in this study are not within the scope of this publication.

### A role for SD-MMEJ in the Arabidopsis germ line?

We observed that - independent of the sgRNA and of the genomic locus – DSB repair in somatic cells predominantly results in one bp insertions and is followed by deletions of increasing size. These are typical outcomes of the cNHEJ repair pathway and confirm earlier reports (Fauser *et al.* 2014, Feng *et al.* 2014). We also observed that for certain sgRNAs a relatively larger deletion of a particular size (7-14 bp) was more frequently observed than for other sgRNAs in somatic cells. These deletions are likely the result of alt-EJ-mediated repair that takes place in somatic cells, albeit at lower low levels. Remarkably, inherited mutations often showed hallmarks of alt-EJ. More precisely, we could show that two alleles studied here fulfilled all criteria for the model of SD-MMEJ repair. This model explains the presence of templated insertions in *grxs17-3* and *grxs17-4*. The combination of deletions with insertions (“filler DNA”) for DSB repair in plants was first reported in tobacco (Gorbunova & Levy 1997; Salomon & Puchta 1998). More recently, a related observation in Arabidopsis called microhomology-mediated synthesis-dependent strand annealing (MM-SDSA) was reported when studying CRISPR/Cas9-mediated DSB repair in regions with homology in somatic cells (Vu *et al.* 2017a, Vu *et al.* 2017b). However, while in MM-SDSA longer deletions and insertions were studied and reported (averaging 240 bp for deletions, Vu *et al.* 2017a), 30 bp has been proposed for SD-MMEJ as a limit of the P1 sequence from the cut site. This was rather arbitrarily chosen as it corresponds to the binding capability of TRIMERIC REPLICATION PROTEIN A (RPA) that binds and protects single stranded DNA (Khodaverdian *et al.* 2017). Moreover, the TIDE method used in our study limits detection up to 50 bp deletions. Therefore, it is unclear at the moment if MM-SDSA and the SD-MMEJ are the same or molecularly related. PolQ has been suggested to mediate SD-MMEJ and was essential for templated insertions in human cells (Yu and McVey, 2010, Saito *et al.* 2017). The recent identification of Arabidopsis PolQ (van Kregten *et al.*, 2016) may allow studying the role for PolQ in SD-MMEJ in the Arabidopsis germline. However, as polQ mutants are resistant to T-DNA integration (van Kregten *et al.*, 2016), they could not be readily used in the workflow we present here to test the involvement of polQ in SD-MMEJ in Arabidopsis.
