# Corrigendum

**DOI:** 10.1534/g3.119.400709

**Published:** 2019-10-29

In the article by L. Pauwels, R. De Clercq, J. Goossens, S. Iñigo, C. Williams, M. Ron, A. Britt, and A. Goossens (*G3: Genes|Genomes|Genetics* 8: 2603-2615) entitled “A Dual sgRNA Approach for Functional Genomics in *Arabidopsis thaliana*” on page 2613 in the first sentence of the Acknowledgments section, incomplete funding information was provided related to support from Research Foundation Flanders. The sentence has been corrected as follows:

This work was supported by the Research Foundation Flanders through the projects 1507013N, G005212N, G005312N and G005915N and a postdoctoral fellowship to L.P.; the Belgian Science Policy Organization for a postdoctoral fellowship to S.I. and the Agency for Innovation by Science and Technology in Flanders for a predoctoral fellowship to J.G.; the National Science Foundation for M.R.’s contribution to this work by grant # 1636397.

